# Focal Xanthogranulomatous Pyelonephritis with Pulmonary Lesions on the Background of Type Two Diabetes Mellitus

**DOI:** 10.1155/2018/1698286

**Published:** 2018-01-31

**Authors:** Ahmad Enshaei, Arash A. Boora, Diana Taheri, Zahra Changizi, Nahid Bahmani

**Affiliations:** ^1^Isfahan University of Medical Science, Kashani Hospital, Kashani Ave, Isfahan, Iran; ^2^University of Queensland, St. Lucia, QLD, Australia; ^3^Department of Pathology, Isfahan Kidney Diseases Research Centre, Isfahan University of Medical Sciences, Isfahan, Iran; ^4^Department of Internal Medicine, Saadi Hospital, Isfahan, Iran; ^5^Department of Radiology, Saadi Hospital, Isfahan, Iran

## Abstract

Focal Xanthogranulomatous pyelonephritis is a rare chronic inflammatory condition of kidneys which usually is associated with postrenal obstruction or renal stone leading to chronic bacterial infection and eventually chronic glomerular inflammation. About 90% of cases are of the diffuse type and associated with staghorn renal calculi. The case presented in this paper is of the focal type in a 58-year-old diabetic female. Interestingly she did not have symptoms or laboratory presentation of chronic renal bacterial infection except for elevated ESR. She sought medical attention due to severe pulmonary infection of the background of morbid obesity. Imaging studies revealed several pulmonary lesions and a large mass of the right kidney which was indistinguishable from renal malignancy. After surgical resection of the right kidney, the lesion is pathologically diagnosed to be a focal Xanthogranulomatous pyelonephritis. The pulmonary lesions were spontaneously resolved about three months following right nephrectomy.

## 1. Introduction

Xanthogranulomatous pyelonephritis (XGP) is a chronic inflammatory renal condition [1]. It is manifested in three types: diffuse (83%–90%), segmental, and focal (together 10%–17%) [2]. The pathogenesis of the disease constitutes lipid-laden macrophages replacing the renal parenchyma [3, 4]. The focal form has a reputation for imitating more serious pathologies [5]. Symptomatically XGP presents with urinary tract infection resistant to antibiotics, fever, haematuria, dysuria, abdominal pain, a palpable mass, anorexia, and weight loss [6]. The aetiology is uncertain. However, association with* E. coli* and Proteus Mirabilis urinary infection has been shown [7, 8]. Also, XGP has been linked to obstruction of the renal tract by infected calculi [9–12]. XGP has been presented as a complication of renal transplant [13]. In addition, XGP has been correlated with metabolic syndrome and diabetes mellitus.

## 2. Case Report

A 58-year-old female presented to infectious disease department of the hospital with a severe pulmonary infection on the background of morbid obesity (BMI 43) and type 2 diabetes mellitus for the last 17 years which was under treatment with insulin in addition to hypertension and hyperlipidaemia. She has past surgical history of cholecystectomy. Recently she has lost 25 kg using Liraglutide (Victoza) injections. She weighed 110 kg at the time of admission.

She has no urological symptoms; no flank pain, dysuria, or frequency or gross haematuria, and neither had she reported experiencing any of these symptoms in the past. She had an elevated ESR for a long time with unknown cause. Urine analysis revealed microscopic haematuria. Besides treatment for pulmonary infection, work-up has been initiated to find the cause of elevated ESR and microscopic haematuria.

**Figure 1:**
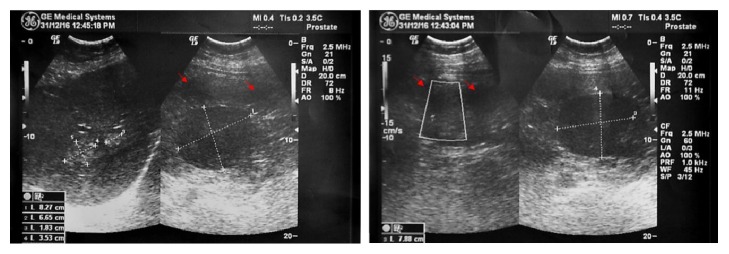
The right kidney masses probably are liver hematomas (red arrows).

An ultrasonography of abdomen and pelvis was performed which revealed a large round hypoechoic solid appearing mass at the lower pole of the right kidney. The mass is virtually indistinguishable from a renal malignancy. The ultrasonography of her abdomen and pelvis was otherwise unremarkable except for evidence of previous cholecystectomy and two small lesions in the liver suspected to be hemangiomas. Then abdominal Computed Tomography with and without administration of contrast medium injection was performed to further investigate the lesion visualized by ultrasonography (Figure 1). A 10 cm × 8 cm heterogeneous soft tissue mass in the lower pole of the right kidney was reported. The mass had faint enhancement and adjacent fatty stranding and pararenal facial thickening.

**Figure 2:**
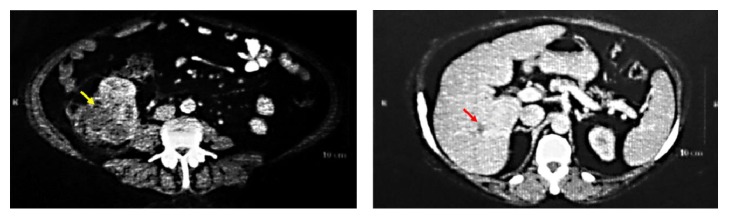
CT of Abdomen. Yellow arrow: mass attached to the right kidney (yellow arrow) on CT scan with bear paw sign. Red arrows: probably liver hemangiomas.

Also, bear's paw sign was observed due to dilation of the renal calyces on CT of the abdomen (Figure 2). Complex cystic renal mass or renal malignancy and cystic degeneration were mentioned as a probable diagnosis.

**Figure 3:**
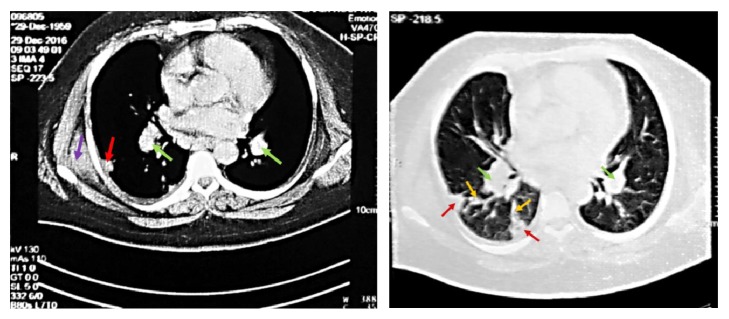
CT of Chest. Lung lesions. Hilar adenopathies (green arrows), probable septic embolization (red arrows), feeding vessels (yellow arrow), and serratus anterior muscle (purple arrow).

In addition, chest X-ray revealed mild pleural effusion and a soft tissue density pleural based lesion in right hemithorax. A thoracic CT scan with and without contrast medium injection was advised to investigate the latter findings further (Figure 3).

Thoracic CT revealed bilateral smaller than 2 cm irregular bordered nodules in both lung fields. Also, a 3.5 cm × 2.5 cm cavitating lesion in the right upper lobe, a wedge-shaped consolidation in the right lower lobe, right pleural effusion, and right hilar adenopathy were seen in thoracic CT scan. Thoracic metastasis was suggested.

On laboratory investigations before treatment, other than elevated ESR (99 in 1 hour), urinalysis shows protein, glucose, and blood in a turbid sample. Urine culture was negative; therefore antibiotics were not administered. Biochemistry shows low sodium (130 normal range: 136–145). Complete blood count shows normocytic anaemia (Hb: 10.3 normal range: 12–17) and slightly elevated white cell count (10.1 normal range 4–10). Tumour markers, CEA, CA19-9, CA125, and CA15-3, were not elevated.

With regard to the imaging findings, renal malignancy (Figure 2) with pulmonary metastasis (Figure 3) was suggested. After consultation with the urologist, the patient was scheduled for right radical nephrectomy. In semiflank position (mild elevated patient right flank), with transperitoneal subcostal incision, classic right radical nephrectomy was performed. Nephrectomy is recommended in patients with an irretrievably impaired kidney due to symptomatic, chronic infection, calculus disease, or severe traumatic injury [14]. The mass had severe adhesions to adjacent organs which were released during operation. The entire mass was sent for pathologic examination.

After operation (right nephrectomy) the patient is in good condition without fever and pulmonary symptoms. Also, ESR is reduced to 65 mm/hr. White cell count was reduced to 7.1 and is within the normal range. It seems that her pulmonary nodules had been septic pulmonary embolisms. There is a similar case presentation in the literature which reports XGP complicated with pulmonary embolism [6]. Our case is the second presenting with this complication.

**Figure 4:**
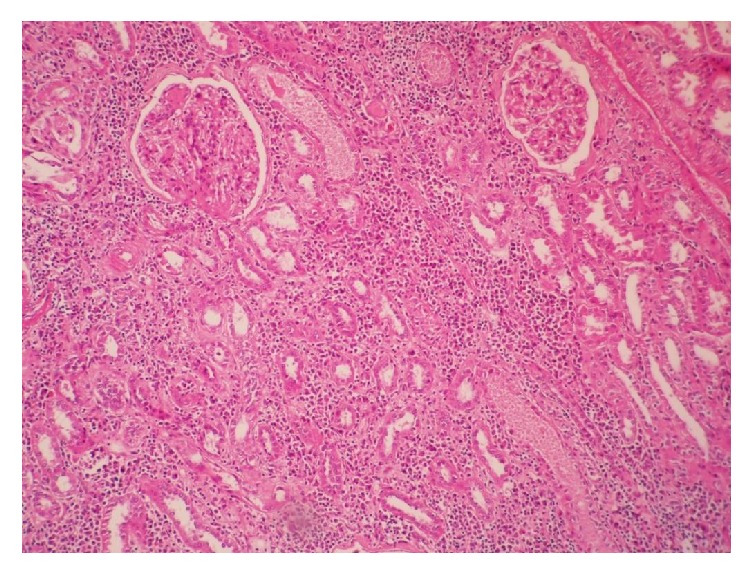
There is marked mixed inflammation in renal parenchyma in the background of diabetic nephropathy (nodular sclerosis). H&E 100x.

On pathological examination, Xanthogranulomatous pyelonephritis (XGP) was diagnosed. On microscopic examination, there is the focal replacement of renal parenchyma by severe mixed inflammatory cells infiltration including lymphoplasma cells, neutrophils and foamy histiocytes infiltration, and fibrosis with extension to perirenal soft tissue. Diabetic nephropathy including nodular sclerosis and arteriolar hyalinosis is seen in the background (Figure 4).

Prophylactic antibiotics were administered at surgery. About three months following right nephrectomy, the pulmonary nodules were found to have spontaneously resolved on chest CT scan.

## 3. Discussion

XGP is a renal chronic granulomatous inflammatory process commonly associated with* E. coli* and* Proteus mirabilis* infection; other likely organisms include* Pseudomonas*,* Enterococcus faecalis*, and* Klebsiella* [8]. XGP usually affects middle-aged females and children [9]. Most instances of diffuse XGP develop in the setting of obstruction due to infected renal calculi [9]. One study showed about 34% of the affected individuals have a staghorn calculus [12]. Usually, there is massive destruction of renal parenchyma by the time of diagnosis [12]. Rarely XGP may present as a complication of renal transplant [13].

XGP appears in three forms: diffuse (typical and common form, 83–90%), segmental, and focal forms (rare 10–17%) [2]. In focal form, the disease is located in renal cortex without renal pelvic communication and, in this form, the renal stone may not be seen. The focal XGP is an imitator of renal neoplasm and is a pseudotumour of the kidney and simply may be mistaken as a renal tumour [15].

Symptomatically, XGP usually presents with stigmata of chronic pyelonephritis including flank pain, fever, malaise, reduced appetite, and weight loss [13]. XGP in children may present differently with fever, abdominal and flank pain, and growth and weight retardation [16].

However, large focal XGP can be symptomless and silent. Sometimes it is found as an incidental finding in an abdominal sonogram that is recommended for other reasons [17]. Interestingly, our patient has not had any symptom related to urinary infection in contrast to almost all these patients as already mentioned in literature who have either symptomatic urinary infection or pyuria (60%) and positive urinary culture (90%) in case of a silent urinary infection [18].

CT scan has been substituted by renal angiography as the diagnostic tool of choice [19]. It has several advantages including the demonstration of the extension of the lesion into pararenal tissue and renal stones as the usual original cause [20]. Also in cases with no renal stone visible on CT, obstruction due to malignancy should be considered as the initiating aetiology of obstruction and the following chronic infection and inflammation [21, 22]. Furthermore, there is no imaging modality that can definitely distinguish between focal XGP and renal malignancy.

On pathological examination, macroscopically, the kidney is enlarged which is unilateral in the majority of cases. Renal stones are enclosed by the mass. If a tumour has infiltrated the perirenal tissue adherence to the adjacent structures may be observed, although uncommon fistula formation may be seen in case the tumour invades gastrointestinal tract [23]. Microscopically, the lesion is composed of three layers around a calyx. The inner zone is composed of necrosis, lymphocytes, leukocytes, plasma cells, and macrophages. The middle zone includes vascularized granulation tissue scattered with haemorrhage. The inflammatory cells are mostly lipid-laden macrophages, hence the yellow colour. The outer section of the lesion is recognized by giant cells and cholesterol clefts [24]. The pathophysiology of XGP involves defected processing of infective bacteria by macrophages which present as giant cells. The pathologic cause of accumulation of lipid in macrophages is not fully understood [10].

The main differential diagnosis of XGP is renal malignancy which cannot be differentiated confidently using CT scan alone. But evidence of chronic renal infection increases the likelihood of XGP. Other renal inflammatory conditions including renal parenchymal malakoplakia and megalocytic interstitial nephritis need to be differentiated from XGP based on their histologic characteristics [25].

Due to a high incidence of the destruction of the kidney parenchyma and the majority of unilateral incidence of XGP, the treatment is almost always surgical en bloc nephrectomy which involves removal of all involved tissues and closure of fistulas if developed. However, an interval of antibiotic is necessary to control the local infection prior to surgery [26]. There is a report of nonsurgical management of XGP [27]. Also, an alternative invasive treatment involving surgical drainage and renal artery embolization is introduced [28]. In case the lesion is focal and has not invaded adjacent structures, a partial nephrectomy may be performed [29]. Laparoscopic nephrectomy may be possible depending on the extension of the lesion and involvement of other structures [26].

## 4. Conclusion

A case of Xanthogranulomatous pyelonephritis (XGP) complicated with pulmonary lesions is presented. Considering that there is no imaging modality which can differentiate focal XGP form renal malignancy, it is important to include the diffuse form of XGP as a differential diagnosis particularly on the background of chronic urinary tract infection and obstruction of the urinary tract by renal calculi. Alternatively, diabetes mellitus has been shown to be associated with focal XGP which has no association with renal calculi.
